# A workflow to design new directed domestication programs to move forward current and future insect production

**DOI:** 10.1093/af/vfab014

**Published:** 2021-06-19

**Authors:** Thomas Lecocq, Lola Toomey

**Affiliations:** INRAE, URAFPA, University of Lorraine, Nancy, France

**Keywords:** biodiversity, domestication, insects, integrative workflow

**Implications**

• Insect farming is expected to expand in the near future, but domestication is a long and difficult process which is often unsuccessful. Considering hits and misses from past directed domestications of insects and other species, we here provide a workflow to avoid common pitfalls in directed domestication programs.

• This workflow underlines that it is crucial to find relevant candidate species for domestication. Candidate species must address human need/demand and meet a set of minimal requirements that shape their domestication potential. The domestication potential can be defined through an integrative assessment of key traits involved in biological functions.

• Geographic differentiation of key traits in a candidate species and the maintenance of adaptative potential of farmed populations should also be considered to facilitate domestication and answer to future challenges.

## Introduction

Domestication has irrevocably shaped the history, demography, and evolution of humans. It is a complex phenomenon which can be seen as a continuum of relationships between humans and nonhuman organisms, ranging from commensalism or mutualism to low-level management (e.g., game keeping or herd management) or, even, direct control by humans over resource supply and reproduction (Terrell et al., 2003; Smith, 2011; Larson et al., 2014; Teletchea and Fontaine, 2014; Zeder, 2014, 2015). This continuum should not be seen as an obligatory succession of different relationships, which ultimately always ends by human control over reproduction, for all species involved in a domestication process. For instance, most fish domestications do not involve initial commensal relationships (Teletchea and Fontaine, 2014), and African donkey-owners do little to manage reproduction of African wild asses (Marshall et al., 2014). Moreover, it is worth noting that the domestication process 1) does not involve all populations of a particular species (e.g., some fish populations underwent domestication for aquaculture while wild conspecific populations still occur, Teletchea and Fontaine, 2014) and 2) is not irreversible (i.e., feral populations).

The complexity of the domestication process is mirrored by the diversity of past domestication histories. For instance, three main patterns of domestication histories can be identified for animal species: the “domestication pathways” (Zeder, 2012a, 2012b, 2015; Larson and Fuller, 2014; Frantz et al., 2016). The commensal pathway (e.g., dog and cat domestications) does not involve intentional action from humans but, as people manipulate their environment, some wild species are attracted to parts of the human niche, and commensal relationships with humans can subsequently arise for the tamest individuals of these wild species (Zeder, 2012b; Larson and Burger, 2013). Over generations, relationships with humans can shift from synanthropic interactions to captivity and human-controlled breeding (Larson and Fuller, 2014). The prey pathway (e.g., domestications of large herbivorous mammals) requires human actions driven by the intention to increase food resources for humans. The pathway starts when humans modify their hunting strategies into game management to increase prey availability, perhaps as a response to localized pressure on the supply of prey. Over time and with the tamest individuals, these game management evolve in herd management based on a control over movements, feeding, and reproduction of animals (Zeder, 2012a; Larson and Burger, 2013). At last, the directed pathway (e.g., domestication of transport animals, Larson and Fuller, 2014) is triggered with a deliberate and directed process initiated by humans in order to control movement, food supply, and reproduction of a wild species in captive or ranching conditions (Zeder, 2012a). All pathways lead to animal population evolution shaped by new specific selective pressures of the domestication environment (Wilkins et al., 2014). The divergence from wild ancestors further increases for species for which humans reinforce their control over population life cycle while they decrease gene flow between populations engaged in the domestication process and their wild counterparts (Teletchea and Fontaine, 2014; Lecocq, 2019). This control can ultimately result in selective breeding programs or organism engineering (e.g., genetically modified organisms) that are developed to intentionally modify some traits of interest (Teletchea and Fontaine, 2014; Lecocq, 2019).

Around 13,000 years ago, a first wave of domestication happened. It concerned mainly terrestrial vertebrate and plant species that are those dominating the agricultural world today (Diamond, 2002; Duarte et al., 2007). Noteworthy examples of insects involved in this wave include the silkworm (*Bombyx mori*, Lepidoptera) and the honeybee (*Apis mellifera*, Hymenoptera) (see domestication histories reviewed in Lecocq, 2019). Many insect domestication events started recently, in the 20th century (Lecocq, 2019), concomitantly with aquatic species (Duarte et al., 2007; Hedgecock, 2012) and some crop taxa (Leakey and Asaah, 2013), during the so-called new wave of domestication (i.e., refers to the large number of domestication trials since the start of the 20th century). Most domestications of this new wave follow a directed pathway through planned domestication programs (Duarte et al., 2007; Teletchea and Fontaine, 2014; Lecocq, 2019). This new wave has been facilitated by technological advances in captive environment control and animal food production. However, the triggering factor of this wave has been the emergence of new unmet human needs. Indeed, new domestication events appear unlikely when the human needs that could be met by targeted species (e.g., human food supply) are already addressed by wild or already domesticated species (Diamond, 2002; Bleed and Matsui, 2010; Freeman et al., 2015). For instance, many of the recent aquatic species domestications have been triggered by the need to meet the rising human demand for aquatic products while wild fishery catches are no longer sufficient (Duarte et al., 2007). Similarly, bombiculture (i.e., production of bumblebees, Hymenoptera, *Bombus* spp.) is an insect example of domestication triggered by an unmet human demand: the development of fruit production (e.g., tomatoes, raspberry) in greenhouses, which required importing insects such as bees to ensure the pollination ecosystem service. However, previously domesticated species, such as honeybees, are quite inefficient pollinators for such crops whereas bumblebees are ideal pollinators for these plants (Velthuis and van Doorn, 2006). This led to domestication of several bumblebee species since the 1980s (Velthuis and van Doorn, 2006). Overall, for insects, as for many other species, recent domestication programs have been triggered by needs to produce biological control agents (e.g., ladybugs, Coleoptera, Coccinellidae), pets (e.g., hissing cockroach, Blattodea, *Gromphadorhina portentosa*), and laboratory organisms (e.g., fruit flies, Diptera, *Drosophila* spp.), or for sterile insect technique development, and raw material/food production (reviewed in Lecocq, 2019).

New instances of insect domestication can be expected in the near future as several authors and international organizations claim that larger, optimized, and new insect productions will be a part of the solution to ensure human food/sanitary security and to address new demands for pets in the next decades (van Huis et al., 2013; Gilles et al., 2014; Lees et al., 2015; Mishra and Omkar, 2017; Thurman et al., 2017; Saeidi and Vatandoost, 2018). Here, we speculate that these future domestications will mainly follow a directed pathway as observed for other species involved in the new wave of domestication. These future domestication programs will be challenging since, despite technological developments, directed domestication is still a long and difficult process which often ends up being unsuccessful. Even when the life cycle is controlled by humans, major bottlenecks can still hamper the development of large-scale production. Although limited amount of information about domestication failure rate is available in literature, past domestication programs of species involved in the new wave of domestication show that many new domestication programs often lasted a couple of years before being abandoned (e.g., for fish: Metian et al., 2019; for insect: Velthuis and van Doorn, 2006). The main causes of these failures are technical limitations, socioeconomic constraints, or intrinsic species features (Liao and Huang, 2000; Diamond, 2002; Driscoll et al., 2009). Potential solutions to facilitate domestication have been investigated for plants and vertebrates (e.g., Diamond, 2002; DeHaan et al., 2016; Toomey et al., 2020a). Conversely, insects have received very little attention to date (Lecocq, 2019).

**Figure 1.**
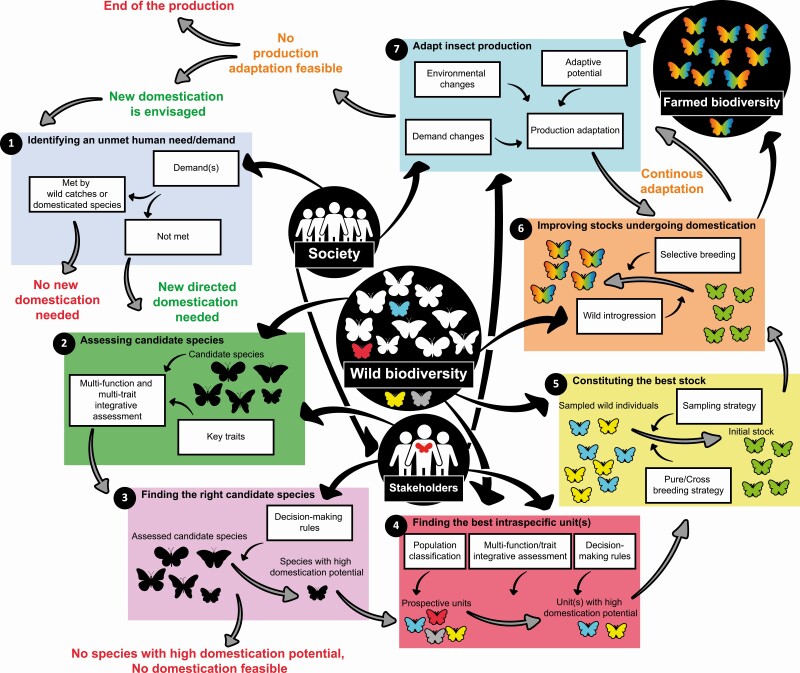
A seven-step workflow to develop a fruitful insect production. 1. Identification of an unmet human demand. 2. Identifying candidate species that could meet the demand through a multifunction and multitrait assessment jointly developed with stakeholders. 3. Decision-making rules established with stakeholders highlight species with high domestication potential (here, one species but several species can be chosen). 4. Investigating the interest of geographic differentiation between wild populations (prospective units) of the species, similar to steps 2 and 3 to highlight units with high domestication potential (two units in this fictive example). 5. Creating the initial stock through pure or cross breeding strategy with attention paid to the genetic diversity of this stock (here, a cross breeding strategy is used). 6. Initial stock improvement through selective breeding programs and/or wild introgression to minimize adverse effects and reinforce beneficial domestication effects. 7. Production evolution according to human demand and environmental changes thanks to its adaptive potential and methods developed in the previous step. When no adaptation can be developed, new domestication could be considered. Wild biodiversity is considered at the species and intraspecific levels.

Here, we consider feedbacks from past directed domestication programs of insects and other species to provide a conceptual workflow (Figure 1) to facilitate future insect domestication programs following a directed pathway (from this point, domestication will refer in the text to the directed pathway). This workflow ranges from the selection in the wild biodiversity of biological units (at species and intraspecific levels) to start new production to the development of selective breeding programs. We considered that technical limitations are not a major issue in insect domestication. Indeed, production systems (i.e., human-controlled environments in which animals are reared and bred) are already available for several phylogenetically distant insect species with different ecology, physiology, and behavior (Leppla, 2008). Thus, future insect domestications could likely be based, with potentially minor adjustments, on already existing production systems. Therefore, we here focus on how avoiding pitfalls due to socioeconomic constraints or intrinsic species features to move forward ongoing and future directed insect domestication programs to response to human demands.

## Backing the Right Horse by Finding the Right Candidate Species for Domestication

Domestication processes which meet needs that can be more easily addressed by other means (e.g., wild catches or other domesticated species), as well as productions with a low productivity and/or profitability, are often doomed to failure (e.g., Diamond, 2002; DeHaan et al., 2016). Therefore, any new planned domestication program should consider how it could respond to an unmet human requirement with a viable and efficient business model. This can be at least partially answered by an evaluation of potential candidate species for domestication before starting large-scale production.

### First: identifying an unmet human need or demand to define new candidate species

Human need or demand can focus on a species of interest (species-targeted domestication). Such domestications happen 1) when a wild species already exploited by humans becomes rare (e.g., for insects see Lecocq, 2019) or protected (e.g., the European sturgeon, Actinopterygii, *Acipenser sturio*) in the wild, 2) to allow reintroduction for wildlife conservation (e.g., for butterflies, Crone et al., 2007), or 3) to develop sterile insect techniques (see Lecocq, 2019). At this stage, the species of interest is regarded as a candidate species that must be further studied to assess the feasibility of its domestication (Figure 1).

The need or demand for a particular ecosystem service can also spark new species farming (service-targeted domestication, see also DeHaan et al., 2016), as exemplified by bumblebee domestication (Velthuis and van Doorn, 2006). Since most ecosystem services can be ensured by numerous taxa, several candidates for domestication could be identified. This raises the need to highlight among available candidates those that maximize the chance of success to go successfully through the domestication process (DeHaan et al., 2016).

### Second: the importance of an integrative assessment of candidate species

Before going any further in the domestication program development, special attention should be paid to international and national regulations regarding sampling, transport, and use of candidate species. Indeed, such regulations can prevent producing or trading a species in some areas (e.g., Perrings et al., 2010; Samways, 2018), making its domestication economically poorly attractive or pragmatically useless. They can thus limit the number of potential candidates or make a species-targeted domestication unfeasible.

Wild insect species are not all suitable candidates for domestication. Indeed, each species has a specific “domestication potential” (adapted from Toomey et al., 2020a): a quantification of how much expression of key traits is favorable for domestication and subsequent production. Several behavioral, morphological, phenological, and physiological key trait expressions have been highlighted as relevant to facilitate domestication and subsequent production (e.g., for noninsects, Diamond, 2002; Driscoll et al., 2009). By considering insect specificities, we state that these expressions include high growth rate, high food conversion ratio, generalist herbivorous feeder or omnivorous, high survival rate, short birth spacing, polygamous or promiscuous mating, large environmental tolerance, high disease resistance, gregarious lifestyle, and diet easily supplied by humans. This list should be completed with additional key traits specific to the domestication purpose. For instance, pollination efficiency is relevant for pollination-targeted domestication while nutritional quality is important for edible insect domestication. Moreover, expression of socioeconomical key traits must also be considered for domestication potential assessment such as high yield per unit, high sale value, established appeal for consumers, and useful byproducts (e.g., for silkworm; Lecocq, 2019). At last, potential environmental consequences of future production, such as risks of biological invasions associated to the development of international trade (Lecocq et al., 2016), should be considered through the evaluation of relevant traits (e.g., invasive potential, which corresponds to the ability of a species to trigger a biological invasion out of its natural range). Overall, the set of key traits can be defined thanks to advice or expectations of stakeholders (consumers, environmental managers, policy makers, producers, and socioeconomists) (Figure 1; see similar approach for fish in Toomey et al., 2020a).

It is worth noting that key traits 1) are involved in different biological functions (behavior, growth/development, homeostasis, nutrition, reproduction) and 2) are not necessarily correlated among each other, implying that expression of a trait cannot be inferred from other traits (Toomey et al., 2020b). This means that species domestication potential must be assessed by a multifunction and multitrait integrative framework (Figure 1). Moreover, species might present specificities in the wild but those might not be maintained in production systems because expression of key traits, as any phenotypic trait, is determined by genetic divergence and environment, as well as the interaction between these two factors (Falconer and Mackay, 1996). Therefore, an efficient assessment should be performed in experimental conditions as close as possible to the production system. Overall, such an assessment can be seen as heavy-going and time- and money-consuming. However, the complexity of multifunction and multitrait assessment in standardized conditions is offset by the minimization of the risk to start a long and difficult domestication program with the wrong candidate species.

### Third: reaching a consensus to choose relevant candidate(s) to start domestication

Making an integrative assessment of domestication potential should not hide the fact that some key traits can be more important than others. For instance, very low survival rate or low reproduction rate during the assessment will certainly stop ongoing domestication trials because they prevent the completion of the life cycle. Therefore, minimal expression threshold (i.e., minimum threshold for a trait expression which must be met or else the biological unit is not suitable for domestication programs; e.g., a survival rate below which an animal production would not be economically feasible) should be defined, potentially by a panel of stakeholders, for the most important traits relatively to the domestication purpose (see similar approaches in DeHaan et al., 2016; Toomey et al., 2020a). When a species does not meet this threshold, it must be regarded to be void of domestication potential. This threshold must be carefully defined, even in species-targeted domestication programs, to avoid starting large-scale domestication programs with issues that could be costly and slow or impossible to fix later in the process.

When comparing key trait expressions between species, it is likely that a candidate displays a favorable expression for a specific key trait (e.g., best nutritional value) but not for another trait (e.g., lowest survival rate). This requires making a consensus between results of key trait assessment to identify the best candidate species for a service-targeted domestication or to objectively assess the relevance of a species-targeted domestication (Figure 1, e.g., for noninsect species, Quéméner et al., 2002; Alvarez-Lajonchère and Ibarra-Castro, 2013; DeHaan et al., 2016). Scoring solutions could be used, considering weighting coefficients to integrate the potential differential levels of importance of key traits due to socioeconomic factors, absolute prerequisites for domestication, or production constraints. Weighting coefficients can be defined through surveys of stakeholders’ expectations (Figure 1; see examples in Quéméner et al., 2002; Toomey et al., 2020a). Since expectations might vary across stakeholders, decision making should be based on a consensus between all parties involved (see strategies to solve complex scientific and socioeconomic issues and consensus solutions in Hartnett, 2011; Wyborn et al., 2019; Toomey et al., 2020a). Ultimately, weighted integrative assessment of candidate species allows highlighting those that would likely foster new fruitful domestication programs for service-targeted domestication or confirm/infirm the relevance of a species-targeted domestication process. These candidates are thus called species with high domestication potential.

## Getting Off on the Right Foot Thanks to Intraspecific Diversity

### Fourth: having the best intraspecific unit to start new domestication programs

Once a new species with high domestication potential has been identified, considering geographic differentiation between allopatric groups of conspecific populations (commonly observed in insects; e.g., Araki et al., 2009; Uzunov et al., 2014) can be helpful to further facilitate domestication programs (Toomey et al., 2020a). Indeed, such population groups can present divergent demographic histories, which can shape genetic and phenotypic specificities through 1) gene flow limitation or disruption, 2) random genetic drift, and/or 3) local adaptation (Mayr, 1963; Avise, 2000; Hewitt, 2001; Toomey et al., 2020a). This could ultimately lead to differentiation in key traits and, thus, to divergent domestication potentials between wild population groups. A few past domestication histories show that geographic differentiation can facilitate domestication (e.g., for fishes: Toomey et al., 2020a; for crops: Leakey, 2012; Leakey et al., 2012). In insects, the domestication of the buff-tailed bumblebee (Hymenoptera, *Bombus terrestris*) is one of the few stunning examples where population-specificity inclusion in domestication programs fostered a fruitful economic development. The buff-tailed bumblebee displays significant differentiation in key traits (e.g., foraging efficiency, colony size, and diapause condition) between differentiated groups of populations corresponding to subspecies (Velthuis and van Doorn, 2006; Kwon, 2008; Lecocq et al., 2016). In the early years of production, European bumblebee breeders tried to domesticate several subspecies. Within a short space of time, one subspecies (*B. terrestris dalmatinus*) proved to have superior characteristics from a commercial point of view (i.e., largest colonies, efficient highest rearing success rate, high pollination efficiency) and became the dominant taxa in the bombiculture industry (Velthuis and van Doorn, 2006). Similarly, non-African honeybees were favored for domestication and production due to facilitating key traits (e.g., low tendency to swarm, survival in temperate areas, low aggressiveness) for beekeeping over African honeybees (Wallberg et al., 2014).

Potential importance of geographic differentiation for insect domestication programs raises the question about how it should be integrated in domestication processes. To this end, a new integrative approach has been recently developed for fish domestication (see Toomey et al., 2020a). This approach provides an integrative assessment of differentiated allopatric population groups through three steps (Figure 1). The first step aims at classifying wild populations of a targeted species in prospective units through phylogeographic or systematic methods. These units are groups of allopatric populations that are likely differentiated in key trait expressions. The second step provides an integrative multifunction and multitrait assessment, similar to interspecific comparison of domestication potential but applied to prospective units. Finally, the last step highlights prospective units with higher domestication potentials (so-called units with high domestication potential, **UHDP**) through the calculation of a domestication potential score through the help/advice from stakeholders (see Toomey et al., 2020a).

### Fifth: constituting the best stock to start new domestication programs

When several UHDP are highlighted as of interest, the question can be raised regarding which strategy should be adopted to constitute the initial stock (Figure 1): 1) keeping only one UHDP or breeding several UHDP apart (“pure breeding” strategy) or 2) mixing UHDP (“cross breeding” strategy) (Falconer and Mackay, 1996). Pure breeding consists of starting with one biological unit and continuously improving it through time (e.g., for *B. terrestris*, Velthuis and van Doorn, 2006 or *A. mellifera*, Uzunov et al., 2014). It is an effective strategy when one biological unit presents a much higher domestication potential than others. In contrast, crossbreeding could be an interesting alternative (e.g., see trials with tasar silkworm, Lepidoptera, *Antheraea mylitta*, Lokesh et al., 2015) when several units present a similar domestication potential or complementary interests. It consists of crossing two or more biological units aiming at having progeny with better performances than parents through complementary of strengths of the two parent biological units and heterosis (i.e., hybrid vigor). However, it is a hit-or-miss strategy since results are hardly predictable (e.g., negative behavioral consequences in *A. mellifera* crossings, Uzunov et al., 2014). The choice regarding which strategy should be used must made on a case-by-case basis.

Further attention should be paid to genetic diversity when constituting the initial stock (Figure 1). If this stock is constituted with a low number and/or closely related individuals, the resulting low global genetic diversity of farmed populations will quickly lead to inbreeding issues, which can be especially damaging in some insect groups such as Hymenoptera (Gerloff and Schmid-Hempel, 2005). It is even more important in the pure breeding strategy which most likely leads to a lower initial genetic diversity than cross breeding approaches. Therefore, care should be taken that a sufficient number of individuals/families (i.e., sufficient effective size) is considered (i.e., sampling strategy) to 1) have a sufficient initial genetic variability and avoid to sample kin individuals which increase risks of future inbreeding issues, 2) mitigate the risk of sampling suboptimal genotypes which are not representative of the population group, and 3) have a sufficient genetic variability for future selective breeding programs (Toomey et al., 2020a).

## Going Further in the Domestication Process: The Wise Way

### Sixth: improving stocks undergoing domestication

During domestication, farmed populations undergo new selective pressures from the rearing environment, a relaxation of wild environmental pressures, and other genetic processes, such as founder effect, genetic drift, or inbreeding (Wilkins et al., 2014). These processes lead to genetic, genomic, and phenotypic differentiations (Mignon-Grasteau et al., 2005; Wilkins et al., 2014; Milla et al., 2021), which are overall poorly studied in insects compared with other taxa (Lecocq, 2019). Yet, they can trigger changes in key trait expressions that are often observed in domesticated species (e.g., for insects: higher tameness, lower aggressiveness toward humans and conspecifics (Latter and Mulley, 1995; Adam, 2000; Krebs et al., 2001; Zheng et al., 2009; Chauhan and Tayal, 2017; Xiang et al., 2018). These changes can facilitate domestication or lead to an improvement of performances (i.e., beneficial changes) that enhances the profitability of the production sector (e.g., higher silk production in silkworm; Lecocq, 2019). However, some changes can also be unfavorable for domestication and subsequent production (i.e., adverse changes) as shown in other taxa (e.g., reproduction issues in fish, Milla et al., 2021).

Selective breeding programs are widely used as a solution to overcome adverse changes or reinforced beneficial changes shaped by domestication (Figure 1). The efficiency of such programs was demonstrated for several taxa (e.g., broiler chicken, *Gallus gallus domesticus*, Galliformes, Tallentire et al., 2016, Atlantic salmon, *Salmo salar*, Salmoniformes, Gjedrem et al., 2012), including insects (e.g., Adam, 2000; Simões et al., 2007; Zanatta et al., 2009; Bourtzis and Hendrichs, 2014; Niño and Cameron Jasper, 2015). Despite the success of numerous breeding programs, they can also lead to negative-side effects. This is well known in livestock (Rauw et al., 1998) but it was also investigated in insects (e.g., Oxley and Oldroyd, 2010). An alternative solution to solve deleterious changes shaped by domestication relies on introgression of wild individuals in farmed populations (Figure 1, Prohens et al., 2017). For instance, in insects, a hybridization was performed between wild African and domesticated European *A. mellifera* populations to create an Africanized strain which would be better adapted to tropical conditions and present a higher honey production (Spivak et al., 2019). However, despite its efficiency for honey production, its defensive behavior quickly became an issue and is considered nowadays as a matter of concern in Americas (Spivak et al., 2019). Overall, the development of selective breeding programs or wild introgression in insect domestication could be of great interest but attention should be paid to traits selected and to potential negative consequences.

### Seventh: keeping one step ahead by maintaining the adaptive potential of production

The relevance of an insect production depends on the socioeconomic and environmental contexts which can change over time. First, the triggering factor of domestication events, the human demand/need, can change with time and/or additional demands can appear aside from the original ones due to market fluctuations, new regulations, or technological development. Second, ongoing global changes (e.g., global warming, pollution) can impact production systems (i.e., outdoor production) and/or availability of important resources for farming (Decourtye et al., 2019). This places a premium on maintaining the adaptive potential of insect production over time, jointly with stakeholders, through species intrinsic features, selective breeding programs, wild individual introgressions, or new domestication program developments (Figure 1).

Insect farming can face these changes thanks to species intrinsic features such as large climatic tolerance or generalist diet. In the context of global changes, the ability to cope with environmental changes is thus a valuable information that should be considered early in the process, during the assessment of candidate species domestication potential (see examples of species-specific responses to climate change or abiotic parameters between closely related species in (Oyen et al., 2016; Martinet et al., 2020).

Alternatively, insect productions can evolve to deal with socioeconomic and environmental changes through selective breeding programs (i.e., continuous adaptation) to improve farmed populations (through trait selection or wild introgression) or create new specialized strains (Decourtye et al., 2019). However, selective breeding programs often drive to a loss of genetic diversity, which can trigger a lower resilience of farmed stocks (Gering et al., 2019). Indeed, genetic variability defines a biological unit’s ability to genetically adapt to future challenges and contributes to global species biodiversity, which maximizes species survival chances in the long term (Sgrò et al., 2011). This appears even more important considering that some rearing practices can quickly lead to a loss of genetic variability (e.g., beekeepers specializing in queen breeding and consequently a large amount of progeny originate from a few queen mothers, Meixner et al., 2010). Moreover, genetic variability can also be important for the population fitness (e.g., this variability is essential for disease resistance and homeostasis in *A. mellifera*, Meixner et al., 2010). Overall, the maintenance of genetic variability is capital (Figure 1) and could be facilitated by wild introgressions (Prohens et al., 2017).

Finally, in extreme cases in which farmed stocks cannot face/be adapted to new socioeconomic and environmental contexts, it will be necessary to start new domestication programs using new candidates (new wild species or population groups).

## Conclusion

Insect farming is expected to expand in the future but remains challenging because of the difficulty to domesticate new species. We proposed a conceptual workflow to avoid major problems commonly encountered during domestication programs. We underlined the importance of 1) considering how new species production could respond to an unmet human demand with a viable and efficient business model and 2) assessing the domestication potential of candidate species through an integrative assessment. We argued that geographic differentiation between wild populations of a candidate species can be valuable. At last, we emphasized the importance of maintaining the adaptive potential of productions to answer to current and future challenges.

## About the Authors

**Thomas Lecocq**—He is an associate professor (senior lecturer) in animal biology and ecology. He works on functional ecology, ecological niche modeling, systematics, and phylogeography. His current research projects focus on the development of integrative and conceptual approaches to support domestication programs. He explores how intraspecific diversity and interspecific interactions can facilitate domestication process and increase animal production sustainability.

**Lola Toomey**—After obtaining a MSc in marine biology at the University of Western Brittany (France), she received her PhD in agronomy from the University of Lorraine (France). Her PhD research focused on the integration of geographic differentiation in domestication programs to promote aquaculture diversification. Her current research interests include population genetics, phylogeography, domestication, and agronomy.
